# Investigation of light-induced lacrimation and pupillary responses in episodic migraine

**DOI:** 10.1371/journal.pone.0241490

**Published:** 2020-10-30

**Authors:** Marija Zivcevska, Shaobo Lei, Alan Blakeman, Daune MacGregor, Herbert C. Goltz, Agnes M. F. Wong

**Affiliations:** 1 Program in Neurosciences and Mental Health, The Hospital for Sick Children, Toronto, Canada; 2 Department of Ophthalmology and Vision Sciences, University of Toronto, Toronto, Canada; 3 Division of Neurology, Department of Pediatrics, University of Toronto, Toronto, Canada; 4 Division of Neurology, Department of Pediatrics, The Hospital for Sick Children, Toronto, Canada; 5 The Krembil Research Institute, Toronto Western Hospital, Toronto, Canada; 6 Department of Ophthalmology and Vision Sciences, The Hospital for Sick Children, Toronto, Canada; National Eye Centre, UNITED STATES

## Abstract

The purpose of this pilot study was to investigate the light-induced pupillary and lacrimation responses mediated by intrinsically photosensitive retinal ganglion cells (ipRGCs) in migraine. Ten participants with episodic migraine and normal tear production, as well as eleven visually normal controls participated in this study. Following an initial baseline trial (no light flash), participants received seven incremental and alternating red and blue light flashes. Pupillometry recording of the left eye and a 1-min anesthetized Schirmer’s test of the right eye (using 0.5% proparacaine) were performed simultaneously. Intrinsic and extrinsic ipRGC photoactivities did not differ between migraine participants and controls across all intensities and wavelengths. Migraine participants, however, had significantly lower lacrimation than controls following the highest blue intensity. A positive correlation was found between melanopsin-driven post-illumination pupillary responses and lacrimation following blue stimulation in both groups. Our results show that participants with self-reported photophobia have normal ipRGC-driven responses, suggesting that photophobia and pupillary function may be mediated by distinct ipRGC circuits. The positive correlation between melanopsin-driven pupillary responses and light-induced lacrimation suggests the afferent arm of the light-induced lacrimation reflex is melanopsin-mediated and functions normally in migraine. Lastly, the reduced melanopsin-mediated lacrimation at the highest stimulus suggests the efferent arm of the lacrimation reflex is attenuated under certain conditions, which may be a harbinger of dry eye in migraine.

## Introduction

Photophobia is a common and debilitating sensory disturbance characterized by light-induced ocular or cranial discomfort that can be accompanied by a subsequent increase in tear production and squinting responses [1, 2]. The most common neurologic condition associated with photophobia is migraine, with 80%-90% of patients experiencing photophobia [3, 4] both during (ictally) and in between (inter-ictally) migraine attacks [5–7]. Migraine headache is thought to arise from the dural trigeminovascular network, whereby light-induced exacerbation of headache likely represents the interaction between afferent retinal photic signals and the trigeminal nociceptive pathway at the level of the thalamus [8]. There is growing evidence in animal models suggesting that the melanopsin-containing intrinsically photosensitive retinal ganglion cell (ipRGC) provides a significant contribution to the photosensory input in photophobia [8–11].

IpRGCs are a third photoreceptor class that mediate non-image forming visual functions, including photoentrainment of circadian rhythms and the pupillary light reflex [12–14], by integrating both extrinsic (rod/cone-driven) and intrinsic (melanopsin-driven) retinal photoactivity. The ipRGC-mediated pupillary light reflex has been a subject of extensive study in recent years. It has since been established that extrinsic and intrinsic ipRGC activity can be selectively assessed with chromatic pupillometry technique, which uses light stimuli of different wave-lengths, intensities, and durations to selectively activate different photoreceptors in the pupillary light reflex pathway [15, 16]. Generally speaking, a brief pupil constriction that quickly returns to baseline in response to a bright red stimulus represents cone-driven extrinsic ipRGC activity. In contrast, a sustained pupil constriction after the offset of a bright blue stimulus, namely post-illumination pupil response (PIPR), represents melanopsin-driven intrinsic ipRGC activity.

Lacrimation has been described as part of parasympathetic dysfunction (light-headedness, nausea, vomiting, salivation, and rhinorrhea) during the ictal, but not inter-ictal, phase in migraine [17]. Perhaps somewhat paradoxically, a number of studies, including several large population-based analyses, have shown an association between migraine and dry eye disease [18–26], indicating that the afferent and efferent pathways of the lacrimation reflex may be abnormal in migraine. The classic lacrimation reflex begins with ocular surface irritation. Sensory (afferent) input activates the ophthalmic branch of the trigeminal nerve (V1), which synapses at the spinal trigeminal nucleus in the medulla. The efferent parasympathetic fibers, originating from the superior salivatory nucleus (SSN) in the pons, exit the brainstem via the facial nerve (CN VII) and travel to the sphenopalatine ganglion. From the sphenopalatine ganglion, they reach the lacrimal gland via the superior branch of zygomatic nerve [27]. Importantly, in addition to ocular surface irritation, the lacrimation reflex can also be induced by bright light, which serves as another type of nociceptive stimulus. Work in the last decade has shown that the SSN plays a central role in relaying light signals. In addition to sensory input from the ocular surface via V1, the SSN receives photic signals from the retina via hypothalamic nuclei, as well as from ipRGCs via the olivary pretectal nucleus [10, 17, 28], providing the neural circuitry for light-induced lacrimation.

We recently used simultaneous chromatic pupillometry recording and light-induced lacrimation measurement to investigate photo-sensitivity (a phenomenon closely associated with photophobia) in normal participants. We found that these two phenomena are highly correlated, and that both are mediated predominantly by melanopsin-induced ipRGC photoactivity [29]. However, photophobia and light-induced lacrimation, as well as their underlying mechanisms, have not been well-studied in patients with migraine. In the present pilot study, we assessed ipRGC-driven pupil responses and their correlation with light-induced lacrimation in a cohort of patients with migraine.

## Methods

### Participants

In total, 28 participants (8 male and 20 female, aged 22–49 years) were recruited through hospital flyers and headache clinic referrals at the Hospital for Sick Children and Women’s College Hospital in Toronto, Canada. Institutional Research Ethics Board approval and written informed consent were obtained. All potential participants underwent a detailed eye exam to assess visual acuity (Early Treatment Diabetic Retinopathy Study [ETDRS] Chart), color vision (Mollon-Reffin Minimal Color Vision Test), refractive error, ocular motility, slit-lamp examination, and a non-dilated fundus exam. As a preliminary measure to rule out the presence of dry eye, participants were asked if they have experienced dry eye symptoms, including eye stinging, burning, gritty felling, redness, irritation or blurry vision during the last week. Slit-lamp exam was performed with fluorescein to assess tear film breaking-up time (BUT) and cornea staining. Participants also completed a non-anesthetized Schirmer’s test, a standard clinical test used to measure tear volume, whereby a filter paper strip (5 x 35 mm) was placed in the conjunctival sac of each eye before the testing took place [30]. Only participants without symptoms or clinical signs of dry eye disease (i.e., BUT < 10 s, corneal punctate staining, and tear volume less than 8 mm within 5 minutes of strip administration) were included. Other exclusion criteria included contact lens wearers, significant ocular media opacities, anterior segment abnormalities that could compromise iris movement, pupillary abnormalities (miosis, mitosis, tonic pupil, anisocoria), retinopathies, optic neuropathies, history of major eye trauma or intraocular surgery, sleep disorders, as well as any other ocular, neurological, psychogenic conditions or medications that could compromise the integrity of the pupillary light reflex pathway.

Ten participants with episodic migraine with or without aura (6 with aura, 9 females, mean age 34 ± 8.7 years, age range 22–49 years) and 11 visually normal controls (5 females, mean age 25 ± 6.6 years, age range 18–42 years) with no history of migraines or photophobia were included in this study. Control data sample has been previously published [29]. All participants with migraine fulfilled the diagnostic criteria of the International Classification of Headache Disorders III [31] and had confirmed migraine diagnosis by a licensed clinician, with no other neurologic abnormalities or history of traumatic brain injury (see Table 1 for clinical features). All participants with migraine self-reported ictal and inter-ictal photophobia, and experienced no headache symptoms during the testing period. Four of nine female patients reported that their migraine may be triggered by menses. All study protocols adhered to the guidelines of the Declaration of Helsinki.

**Table 1:** Clinical features of migraine participants (n = 10).

Characteristic	Value	Characteristic	Value
**Sex (F/M, %F)**	9/1 (90)	**Unilateral Localization, n(%)**	10 (100)
**Age (mean ± SD)**	34 ± 8.7	**Associated Symptoms, n(%)**	
**Type of Migraine, n(%)**		Nausea	10 (100)
With Aura	6 (60)	Vomiting	6 (60)
Without Aura	4 (40)	Photophobia	10 (100)
**Headache Frequency, n(%)**		Phonophobia	10 (100)
< 4 days/month	7 (70)	**Headache Triggers, n(%)**	
4–7 days/month	1 (10)	Physical Activity	3 (30)
8–14 days/month	2 (20)	Emotional Stress	7 (70)
Headache Intensity*, n(%)		Sensory Stimuli **	10 (100)
Severe	5 (50)	Extended Screen Exposure	6 (60)
Incapacitating	5 (50)	Sleep Disturbances	10 (10)
Headache Duration*, n(%)		Fatigue	8 (80)
4–8 hrs	2 (20)	Menses	4 (40)
9–24 hrs	2 (20)	Alcohol	6 (60)
25–72 hrs	6 (60)	Caffeine	4 (40)
**Headache Pain, n(%)**		Caffeine Withdrawal	4 (40)
Throbbing	10 (100)	Diet	4 (40)
Pressure	4 (40)	Hunger/Fasting	8 (80)
Stabbing	5 (50)	Weather Changes	6 (60)
Dull ache	1 (10)	High Altitude	1 (10)

*Untreated with medication.

**Includes visual, auditory and olfactory stimuli.

### Experimental conditions and procedure

Each experimental session began with a 10 minute dark adaptation period in a quiet and darkened room (0 cd/m^2^ as measured with a Gossen MAVOLUX 5032 C Meter, Nürnberg, Germany, equivalent to 0 log quanta). Participants wore a spectacle frame-mounted eye tracker (Arrington Research, Scottsdale, AZ, USA) that used near-infrared (940 nm) illuminating diodes and mini infrared cameras to record pupil size at a sample rate of 60 Hz. Participants were seated in front of a Ganzfeld stimulator (Espion V5 system with the ColorDome LED full-field stimulator; Diagnosys LLC, Lowell, MA, USA). Long wavelength red (peak wavelength: 635 nm, full width at half maximum: 22 nm) and short wavelength blue (peak wavelength: 470 nm, full width at half maximum: 31 nm) stimuli were used as the light sources. Luminance values were converted to melanopic illuminance and log quanta using the Irradiance Toolbox [32] (see Table 2). All pupillometry recordings were measured in the dark (0 cd/m^2^, as measured with a Gossen MAVOLUX 5032 C Meter, Nürnberg, Germany, which is equivalent to 0 log quanta).

**Table 2:** Stimulus characteristics for light sources used.

Stimulus	Peak λ (nm)	Full Width Half Max	Luminance (cd/m^2^)	Melanopic Illuminance (α-opic lux)	Log Quanta
					(log_10_[1/cm^2^/s])
Red (Long λ)	635	22	10	0.02	13.39
			31.6	0.06	13.81
			100	0.20	14.31
			400	0.80	14.92
Blue (Short λ)	470	31	10	94.82	13.52
			31.6	299.63	14.02
			100	948.21	14.52
			400	3792.85	15.12

At trial onset, topical 0.5% proparacaine eye drops (Alcon, Fort Worth, TX, USA) were administered to anesthetize the right eye. Any residual fluid in the conjunctival sac was absorbed with tissue paper 30 seconds after drop administration, which was followed by placement of a Schirmer’s strip in the inferior fornix. Anesthetization of the right eye served to prevent reflex tear production as a result of mechanical stimulation from the strip, so that light-induced tear volume levels could be measured. Following Schirmer’s strip placement, participants were instructed to quickly position their head on a chin rest in front of the Ganzfeld stimulator and fixate on a central LED fixation target for the duration of the trial.

To assess baseline values, participants received no light stimulation in the first trial. For all the subsequent 14 trials, participants received either a red or blue flash of 400 ms duration 20 s after the trial onset. One minute following the flash, the Schirmer’s strip was gently removed, and the tear volume (mm) was immediately recorded. In total, seven stimulus intensities (0.1, 1, 3.16, 10, 31.6, 100, 400 cd/m^2^) were presented incrementally, in an alternating manner with red first and then blue flashes. There were 15 one-min trials in total. The specifics of this experimental design have been previously reported [29].

### Data processing and analysis

Eye tracker data from the left eye were analyzed offline using a custom-written script (MatLab; MathWorks, Inc., Natick, MA, USA). Blink artifacts were removed using a median (window width of 0.5 second) and low pass (fourth-order, zero-phase Butterworth) filter with a cut-off frequency of 5 Hz. The filtered data were visually inspected using a graphical user interface (GUI) to detect artifacts and maintain data quality.

To account for individual variability, pupil data were normalized to a 5-second baseline period prior to stimulus onset (i.e., normalized pupil size = absolute pupil size/baseline pupil size) and tear volume data were normalized to the baseline recording in trial 1 (i.e., normalized tear volume = light induced tear volume minus baseline tear volume with no light stimulation).

Three variables were measured for this experiment: (1) Maximum Pupil Constriction (MPC)—the smallest pupil size post-stimulus presentation, which represents predominantly rod and cone mediated pupillary dynamics; (2) Post-Illumination Pupillary Response (PIPR)—the mean pupillary constriction over a 20 second interval (10–30 seconds post light stimulation), which serves as a validated index of melanopsin photoactivity [15, 33]; and (3) Tear Production—the length of moisture on a Schirmer’s strip in mm after 1 minute in the conjunctival sac. Previously published control data demonstrate minimal pupillary responses and little tear production relative to baseline for low intensity (0.1–3.16 cd/m^2^) blue and red light stimulation [29]. Therefore, we only report responses to higher intensity stimulation (10–400 cd/m^2^); there was no difference between participants with migraine and controls at low intensities (0.1–3.16 cd/m^2^) for all variables including PIPR (Red: *F*_(1, 19)_ < 0.001, *p* = 0.99, partial η^2^ < 0.001; Blue: *F*_(1, 19)_ = 0.008, *p* = 0.93, partial η^2^ < 0.001), MPC (Red: *F*_(1, 19)_ = 0.18, *p* = 0.67, partial η^2^ = 0.10; Blue: *F*_(1, 19)_ = 0.35, *p* = 0.56, partial η^2^ = 0.02) and Tear Production (Red:: *F*_(1, 19)_ = 2.80, *p* = 0.11, partial η^2^ = 0.13; Blue: *F*_(1, 19)_ = 1.65, *p* = 0.22, partial η^2^ = 0.08).

Statistical analyses of the pupil metrics were computed using SPSS 23.0 (IBM Corporation, Armonk, NY, USA). The dataset contained no outliers, as defined as studentized residuals greater than ± 3. The data passed normality (Shapiro-Wilk test, p > 0.05), covariance (Box’s M test, p > 0.05), and equal variance (Levene’s test, p > 0.05) analyses. In cases where the assumption of sphericity was not met (Mauchly’s test, p < 0.05), the Greenhouse-Geisser adjustment was used. Differences in MPC, PIPR, and tear production were compared using separate two-way mixed ANOVA analyses with two factors: Participant Type (two levels: Migraine and Control) as the between subject factor, and Stimulus Intensity (four levels: 10, 31.6, 100 and 400 cd/m^2^) as the within subject factor. This was done separately for red and blue wavelength data (see Table 3 for descriptive statistics). In the presence of a significant interaction in the two-way mixed ANOVA, simple effects were run, and the data were Bonferroni corrected to adjust for multiple comparisons. The correlation between tear production and PIPR was assessed using Pearson’s correlation coefficient. Statistical significance was set at p < 0.05. There were no statistically significant differences between gender in the control group in all three measured outcome variables, MPC, PIPR, and tear production which is consistent with a previous report [34].

**Table 3:** Descriptive statistics (mean ± SD) of results.

Luminance (cd/m^2^)	Wavelength (λ)	MPC		PIPR		Tear Production	
		Migraine	Control	Migraine	Control	Migraine	Control
10	Long λ	0.40 ± 0.05	0.40 ± 0.05	0.04 ± 0.02	0.02 ± 0.03	2.00 ± 2.01	0.23 ± 2.04
	Short λ	0.52 ± 0.05	0.51 ± 0.05	0.10 ± 0.04	0.07 ± 0.05	3.25 ± 2.92	3.36 ± 3.39
31.6	Long λ	0.42 ± 0.05	0.39 ± 0.06	0.04 ± 0.03	0.04 ± 0.02	1.20 ± 1.03	1.14 ± 1.64
	Short λ	0.57 ± 0.06	0.57 ± 0.06	0.19 ± 0.07	0.18 ± 0.07	3.80 ± 3.86	4.55 ± 4.55
100	Long λ	0.42 ± 0.06	0.41 ± 0.05	0.05 ± 0.03	0.02 ± 0.03	2.65 ± 2.47	1.09 ± 1.43
	Short λ	0.61 ± 0.05	0.61 ± 0.04	0.31 ± 0.09	0.26 ± 0.07	5.40 ± 3.81	8.05 ± 3.26
400	Long λ	0.46 ± 0.05	0.46 ± 0.05	0.05 ± 0.03	0.04 ± 0.03	3.45 ± 2.84	2.95 ± 2.63
	Short λ	0.64 ± 0.05	0.65 ± 0.05	0.44 ± 0.11	0.39 ± 0.10	6.85 ± 4.10	14.05 ± 4.55

## Results

### Light-induced pupillary responses

**Fig 1:**
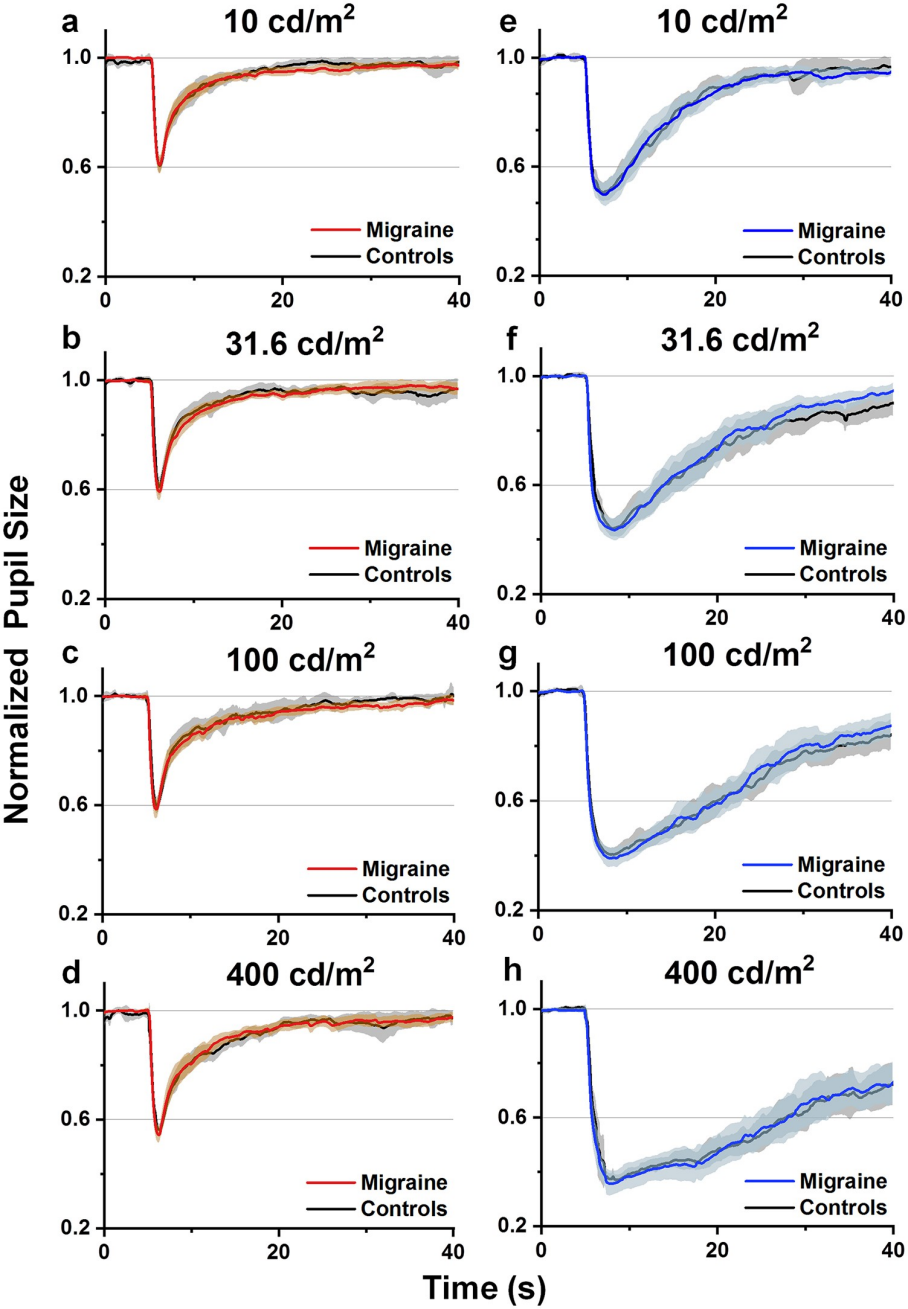
Mean normalized pupillary time courses recorded from the left eyes during simultaneous light-induced lacrimation measurements. Participants with migraine and controls were compared in response to 400 ms (a-d) red and (e-h) blue light stimulation. Error bands represent 95% confidence intervals.

The mean pupillary light reflex responses following red and blue light stimulation at different intensity levels (10, 31.6, 100 and 400 cd/m^2^) are shown in Fig 1. Overall, the pupillary traces overlap between controls and participants with migraine for both red and blue stimuli at all intensity levels.

#### Maximum pupillary constriction (MPC)

**Fig 2:**
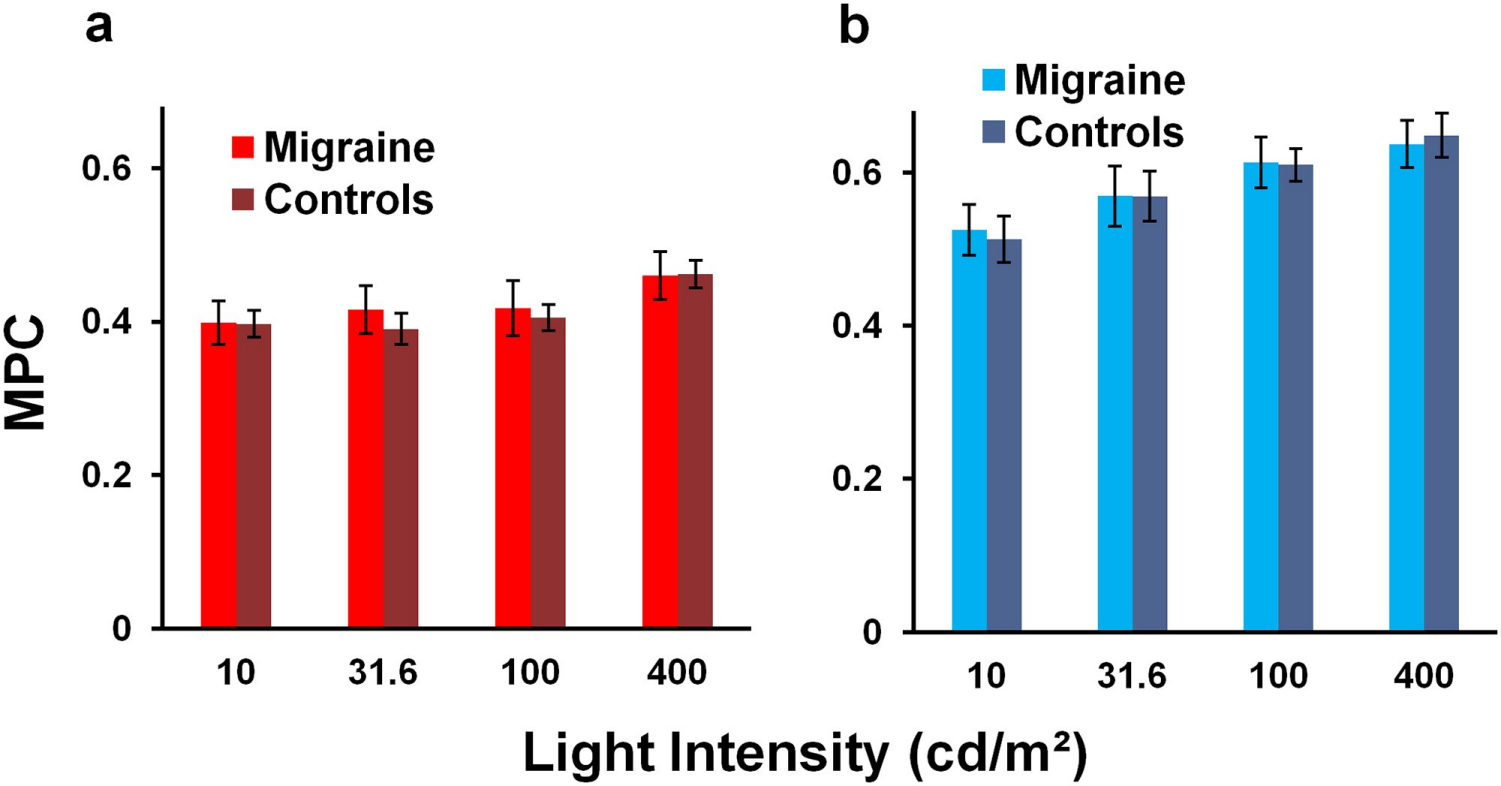
Mean MPC responses of the left eye. MPC was measured in response to four light intensity steps, each of 400 ms in duration, using (a) red and (b) blue light stimulation. Error bars represent 95% confidence intervals. There were no statistically significant differences between the migraine and control groups at each stimulus intensity following red or blue light stimulation.

MPC was not significantly different between the migraine and control groups at any stimulus intensity, whether following red (*F*_(1,19)_ = 0.21, *p = 0*.*65*, partial η^2^ = 0.01) or blue (*F*_(1,18)_ = 0.003, *p = 0*.*96*, partial η^2^ < 0.001) light stimulation (Fig 2). Both wavelengths induced greater MPC responses with increasing light intensity (Red: *F*_(3,57)_ = 23.81, *p < 0*.*001*, partial η^2^ = 0.56; Blue: *F*_(2.22,39.87)_ = 110.38, *p < 0*.*001*, partial η^2^ = 0.86). There was no significant interaction between participants groups and stimulus intensity for both stimulus wavelengths (Red: *F*_(3, 57)_ = 1.04, *p* = 0.38, partial η^2^ = 0.05, Blue: *F*_(2.22, 39.87)_ = 0.97, *p* = 0.40, partial η^2^ = 0.05).

#### Post-illumination pupil response (PIPR)

**Fig 3:**
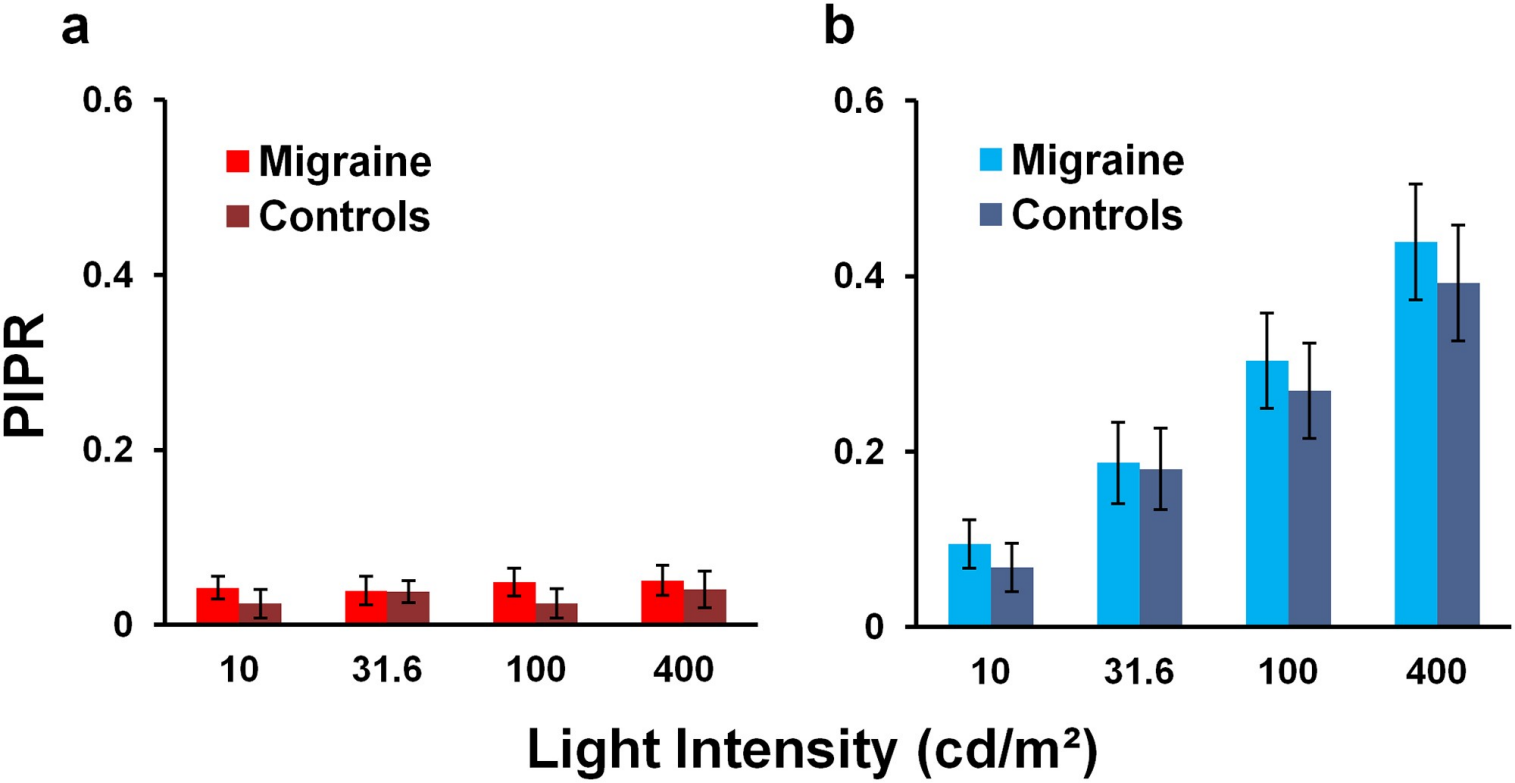
Mean PIPR responses of the left eye. PIPR was measured in response to four light intensity steps, each of 400 ms duration, using (a) red and (b) blue light stimulation. Error bars represent 95% confidence intervals. No statistically significant differences were found between migraine and control groups at each stimulus intensity following red or blue light stimulation.

Similar to MPC, no significant difference in PIPR was found between the migraine and control groups at each stimulus intensity following red (*F*_(1,19)_ = 2.59, *p = 0*.*12*, partial η^2^ = 0.12) or blue (*F*_(1,18)_ = 1.06, *p = 0*.*32*, partial η^2^ < 0.06) light stimulation (Fig 3). PIPR increased with increasing blue light intensity (*F*_(2.14, 38.44)_ = 171.72, *p < 0*.*001*, partial η^2^ = 0.91); however, the same pattern was not seen following red light stimulation (*F*_(2.08, 39.56)_ = 1.46, *p = 0*.*24*, partial η^2^ = 0.07).

### Light-induced lacrimation

**Fig 4:**
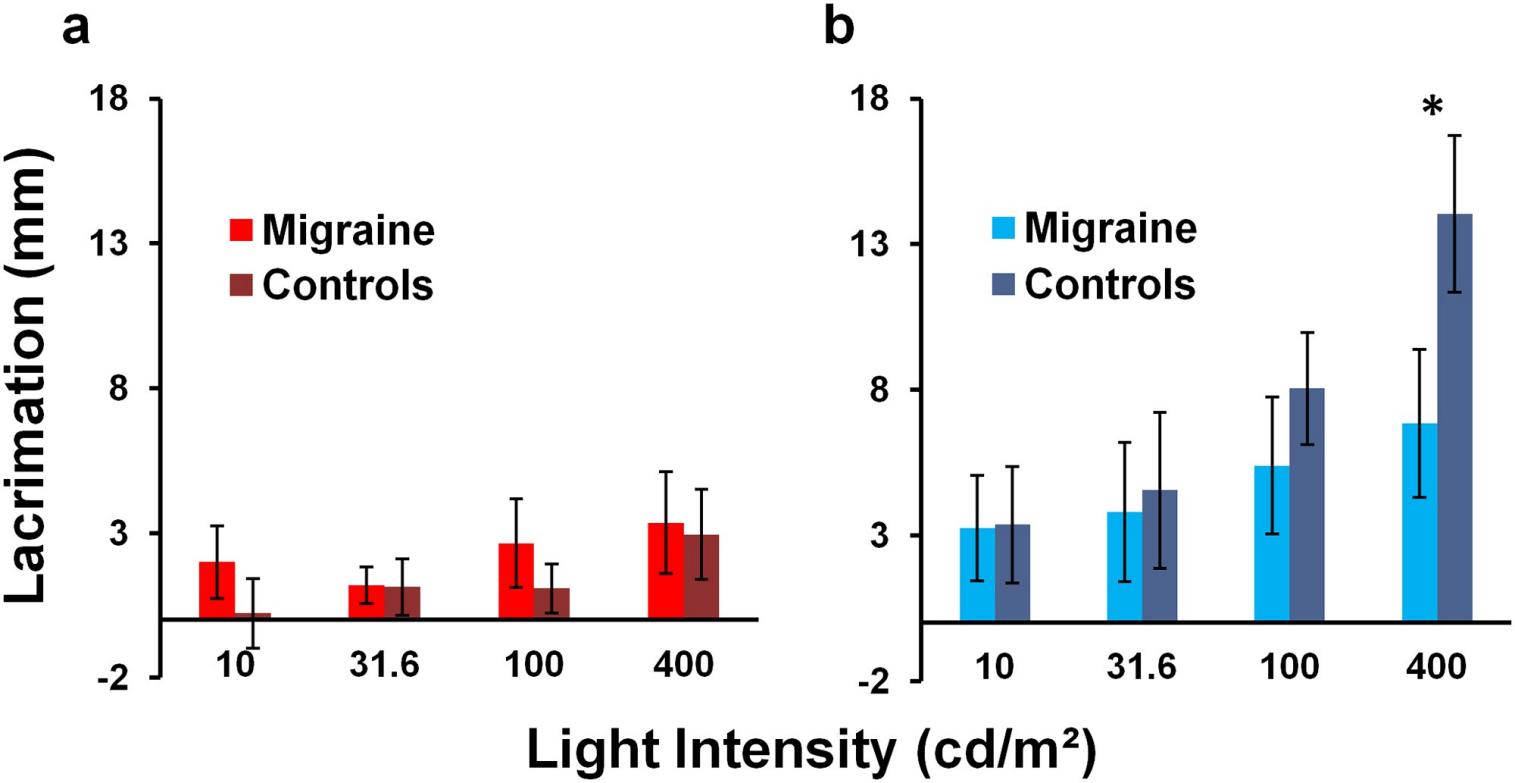
Mean light-induced lacrimation of the right eye. Lacrimation was measured using a 1-min anesthetized Schirmer’s test (mm) in response to four light intensity steps, each of 400 ms duration, using (a) red and (b) blue light stimulation. Error bars represent 95% confidence intervals. The *asterisk* indicates significantly lower tear production in participants with migraine compared to controls at the highest blue light intensity (*p* = 0.001).

Mean tear production in response to chromatic (red vs blue light) stimulation and intensity level are shown in Fig 4. Following blue light stimulation, there was a significant interaction between stimulus intensity and participant type (*F*_(3,57)_ = 9.35, *p < 0*.*001*, partial η^2^ = 0.33); accordingly, simple main effects were computed and corrected for multiple comparisons. Participants with migraine had significantly less tear production than controls following blue light stimulation at 400 cd/m^2^, the highest intensity tested (*F*_(1, 19)_ = 14.34, *p* = 0.001, partial η^2^ = 0.43). No difference between participants with migraine and controls was found at all lower blue light intensity levels (10–100 cd/m^2^).

Within group comparisons show that in participants with migraine, the highest blue light intensity level (400 cd/m^2^) induced significantly greater tear production relative to the lowest intensity levels presented (post hoc pairwise comparison, 400 vs 10 cd/m^2^: *p* = 0.04; 400 vs 31.6 cd/m^2^: *p* = 0.008). No difference was found between the highest intensity level (400 cd/m^2^) and the intermediate level (100 cd/m^2^). For controls, however, 100 and 400 cd/m^2^ intensity levels induced significantly greater tear production relative to the lowest intensity levels (post hoc pairwise comparison, 100 vs 10 cd/m^2^: *p* = 0.006; 100 vs 31.6 cd/m^2^: *p* = 0.04; 400 vs 10 cd/m^2^: *p* < 0.001; 400 vs 31.6 cd/m^2^: *p* = 0.001). The highest intensity level (400 cd/m^2^) also induced significantly greater tear production relative to the intermediate level (100 cd/m^2^; post hoc pairwise comparison, 400 vs 100 cd/m^2^: *p* = 0.006).

Following red light stimulation, there was no significant interaction between stimulus intensity and participant type (*F*_(1.73,32.78)_ = 1.65, *p = 0*.*21*, partial η^2^ = 0.08). No difference in tear production was found between participant types (*F*_(1, 19)_ = 1.80, *p* = 0.20, partial η^2^ = 0.09). Only the highest intensity level (400 cd/m^2^) induced greater tear production relative to the lowest intensity (10 cd/m^2^, post hoc pairwise comparison, *p* = 0.02).

### Correlation between light-induced lacrimation and PIPR

**Fig 5:**
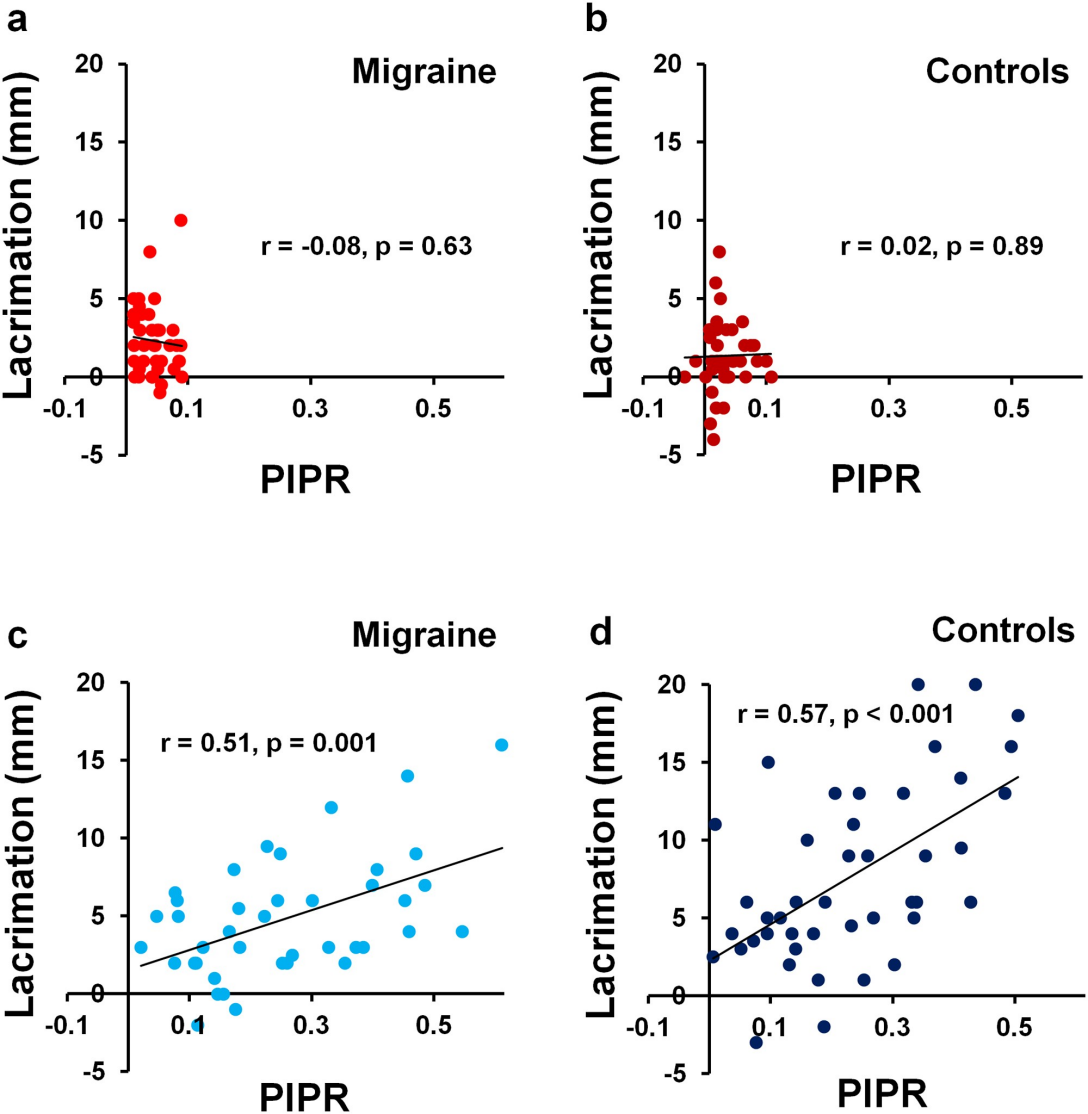
Correlations between light-induced lacrimation and PIPR responses. Raw data from individual trials was used, following red light stimulation for (a) participants with migraine and (b) controls, and following blue light stimulation for (c) participants with migraine and (d) controls.

Person product-moment correlation analysis on all individual trials demonstrated a positive linear correlation between PIPR and tear production for participants with migraine (Pearson’s r = 0.51, p = 0.001, n = 40, Fig 5c) and controls (Pearson’s r = 0.57, p < 0.001, n = 43, Fig 5d) was found in response to blue light stimulation. No significant correlation was found for participants with migraine (Pearson’s r = -0.08, p = 0.63, n = 40, Fig 5a) or controls (Pearson’s r = 0.02, p = 0.89, n = 44, Fig 5b) in response to red light stimulation.

## Discussion

Using chromatic pupillometry to assess the ipRGC-mediated pupillary light reflex pathway, our pilot study demonstrated normal extrinsic (as measured by MPC) and intrinsic (as measured by PIPR) ipRGC activity in migraine patients with photophobia. Our findings suggest that photophobia seen in migraine is unlikely to stem from a hypersensitized ipRGC pathway at the level of the retina (i.e., increased gain), but rather downstream of that circuit.

Three distinct circuits involving retinal afferents have been proposed previously for photophobia: (1) classic rod and cone projections to pain-sensing oculo-vascular trigeminal afferents that ultimately project to the thalamus; (2) direct ipRGC projections to the thalamus; and (3) ipRGC projections to the olivary pretectal nucleus (i.e., part of the pupillary light reflex pathway) [1]. The normal PIPR and MPC we observed provide evidence that the photophobia circuit in migraine is likely distinct from that of the pupillary light reflex. Although both circuits arise from a common afferent origin i.e., the ipRGCs, the photophobia circuit likely involves direct projections from ipRGCs to the thalamus (i.e., the second proposed photophobia circuit), whereas the pupillary light reflex involves projections from ipRGCs to the olivary pretectal nucleus and from there to the Edinger-Westphal nucleus, which gives rise to parasympathetic fibers in the oculomotor nerve (III). Our results lend indirect support to the second proposed photophobia circuit and are in keeping with a mechanism of light-induced exacerbation of migraine headache as proposed by Noseda and colleagues [8] who demonstrated that, in an animal model, ipRGCs directly converge on thalamic trigeminovascular neurons that relay nociceptive signals from the dura to the somatosensory, visual, and associative cortices. It should be noted, however, that because of the redundant and parallel nature of many brain networks, it is possible that all three proposed photophobia circuits are interconnected and interact with each other, and that as yet unidentified circuits may also play a role in photophobia.

Cortez and colleagues recently investigated pupillary dynamics in migraine patients with differing severity and chronicity using a white light stimulation paradigm [35]. They found no inter-ictal group differences in pupil responses between migraine participants and controls, consistent with our findings. They did, however, find that in subsets of patients (i.e., those with chronic migraine and those with most severe migraine profiles), pupil constriction latency and re-dilation time were significantly decreased, suggesting both parasympathetic and sympathetic dysfunctions. The discrepancy in findings between their and our present study is likely the result of differences in patient selection and testing conditions. Further studies with a larger sample size using red and blue light stimulation are warranted to investigate whether photophobia circuits are affected differentially in subgroups of migraine patients with differences in chronicity and severity.

A second important finding in our study is that with the exception of the highest light intensity level, light-induced lacrimation is normal in participants with migraine and that it is correlated with intrinsic melanopsin-driven PIPR (r = 0.51), similar to controls (r = 0.57). These findings collectively indicate that the afferent arm of the light-induced lacrimation reflex pathway is melanopsin-mediated and that it functions normally in participants with migraine. Although it is plausible that the newly identified melanopsin-containing trigeminal fibers [36–38] may contribute to the light-induced lacrimation we observed, their contribution was eliminated or minimized during our experiment because the ocular surface was anesthetized with the use of topical proparacaine, which acts as an antagonist to voltage-gated sodium channels.

A third significant finding in our study is the intriguing observation that light-induced lacrimation is significantly reduced in participants with migraine at the highest light intensity level (>50% less than normal controls), which may shed important light on the possible mechanisms of dry eye disease in migraine. There is a growing body of research showing a high prevalence of dry eye disease in migraine [18–21, 23–25]. Two possible mechanisms have been proposed: (1) neurogenic inflammation of the ocular surface, similar to neurovascular inflammation of cerebral and extracerebral vessels that has been linked to migraine attacks [18, 19, 24, 25]. A mouse model also suggested a link between chronic inflammation, innervation, and altered lacrimal gland function [39]; and (2) anatomical and physiological dysfunctions of the afferent trigeminal pathway e.g., decrease in nociceptive corneal nerve fiber density [22, 26]. At present, it is unclear whether dry eye disease contributes to the pathogenesis of migraine, or whether it represents a consequence of migraine.

What is most notable in the current debate is that it focuses on the *afferent* arm of the lacrimation reflex pathway, while the role of the *efferent* arm has not been investigated. A mechanism by which the ipRGC-mediated pupillary light pathway converges on the parasympathetic *efferent* arm of the classic lacrimation reflex pathway has been proposed by Okamoto and colleagues [28]. They demonstrated that the olivary pretectal nucleus, which receives input primarily from ipRGCs, relays the photic signal to the SSN. The SSN, in turn, stimulates the lacrimal gland through its projection to the zygomatic branch of the facial nerve (CN VII) [28]. They also demonstrated that light-induced lacrimation is reduced when the olivary pretectal nucleus and/or the SSN are pharmacologically blocked. The normal PIPR (i.e., normal afferent arm—see Discussion above) and the reduction in light-induced lacrimation at the highest light intensity level in our participants with migraine suggest that migraine may be associated with an attenuated parasympathetic efferent arm of the lacrimation reflex under certain conditions.

In addition to the melanopsin-dependent retino-midbrain-parasympathetic pathway (i.e., ipRGCs-olivary pretectal nucleus-parasympathetic pathway) described above, another pathway—the retino-hypothalamic-parasympathetic pathway—may also play a role in the light-induced lacrimation reflex. Expanding the scope of previously described central ipRGC projections [40], Noseda and colleagues [17] identified retinal ganglion cell projections to hypothalamic neurons (including the paraventricular, lateral, perifornical, and anterior hypothalamic nuclei, as well as in the ventrolateral preoptic area and periaqueductal gray), which in turn project to the SSN. They proposed that this retino-hypothalamic-parasympathetic pathway is responsible for light-induced exacerbation of parasympathetic symptoms during the ictal phase of migraine. Most relevant to our present discussion, however, is their finding that the effects of this pathway are not influenced by stimulus wavelength and are thus not melanopsin-dependent. We therefore believe that this pathway has minimal or no contribution to the melanopsin-dependent light-induced lacrimation we observed in our experiment.

What is the clinical significance of our findings? It should be emphasized that although we did not perform comprehensive testing to rule out dry eye disease completely, our participants with migraine do not have diagnosable dry eye disease: they did not report any dry eye symptoms and they had a normal (unanesthetized) Schirmer’s test as part of the inclusion criteria. The normal light-induced lacrimation we observed during the inter-ictal phase (except for at the highest light intensity) may correspond to a normal tearing response under most lighting conditions encountered in daily life, which is in keeping with the fact that these patients do not experience dry eye symptoms. However, during the ictal phase when their sensitivity to light is heightened, as simulated by the highest light intensity in our experiment, the reduced lacrimation may reflect a fatigability in reflex tearing or an adaptive process to avoid excessive tearing, mediated by an attenuated parasympathetic efferent response of the lacrimation reflex. It has been proposed that prolonged reduction in parasympathetic tone may cause lacrimal gland hypo-secretion, giving rise to dry eye [41]. The attenuated parasympathetic efferent we observed may be a harbinger of dry eye disease and may explain the high prevalence of the disease, especially in patients with chronic migraine. Although the results from this study are drawn from a small sample, nevertheless, future research using standard criteria of chronic migraine, with special attention to the heretofore neglected role of the efferent parasympathetic arm, are warranted.

## Supporting information

S1 Fig Full dataset of pupillary time courses recoded simultaneously from the left eye of 10 participants with episodic migraines. Pupil responses were compared following baseline (no light flash), (a) blue light stimulation and (b) red light stimulation conditions across various light intensity levels (0.1–400 cd/m^2^).
(TIF)
