# Supplementary figures and images for: Investigation of light-induced lacrimation and pupillary responses in episodic migraine
Source: PLoS One. 2020 Oct 30;15(10):e0241490. doi: 10.1371/journal.pone.0241490 (PMC7598498; doi:10.1371/journal.pone.0241490)

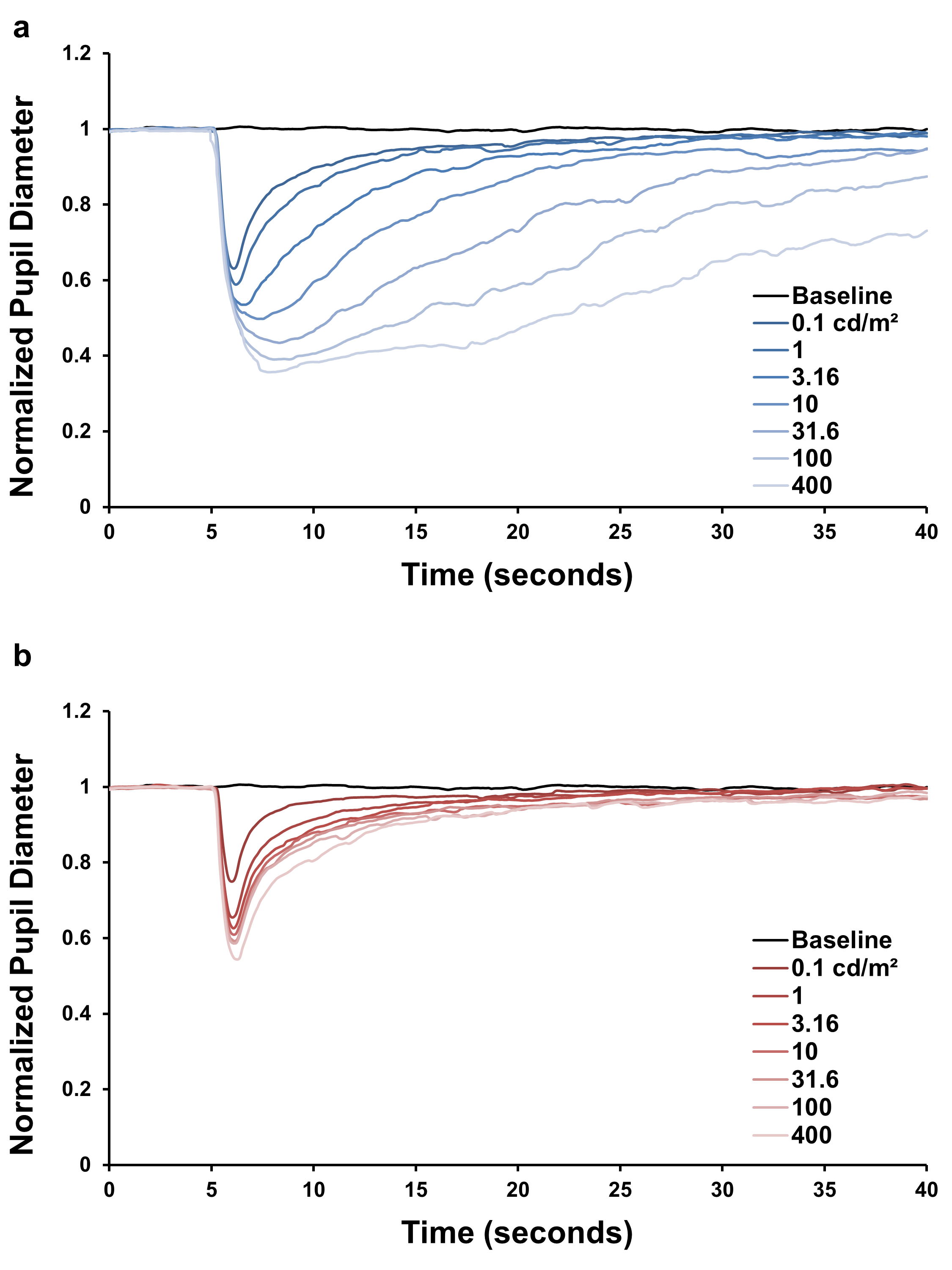

Supplement: S1 Fig — Pupil responses were compared following baseline (no light flash), (a) blue light stimulation and (b) red light stimulation conditions across various light intensity levels (0.1–400 cd/m2). (TIF) [file pone.0241490.s001.tif]
